# Targeting the honey bee gut parasite *Nosema ceranae* with siRNA positively affects gut bacteria

**DOI:** 10.1186/s12866-020-01939-9

**Published:** 2020-08-17

**Authors:** Qiang Huang, Jay D. Evans

**Affiliations:** 1 Honeybee Research Institute, Jiangxi Agricultural University, Zhimin Avenue 1101, Nanchang, 330045 China; 2 USDA-ARS Bee Research Laboratory, BARC-East Building 306, Beltsville, MD 20705 USA

**Keywords:** Honey bee, *Nosema ceranae*, Metatranscriptomics, Bacteria, siRNA

## Abstract

**Background:**

Gut microbial communities can contribute positively and negatively to host health. So far, eight core bacterial taxonomic clusters have been reported in honey bees. These bacteria are involved in host metabolism and defenses. *Nosema ceranae* is a gut intracellular parasite of honey bees which destroys epithelial cells and gut tissue integrity. Studies have shown protective impacts of honey bee gut microbiota towards *N. ceranae* infection. However, the impacts of *N. ceranae* on the relative abundance of honey bee gut microbiota remains unclear, and has been confounded during prior infection assays which resulted in the co-inoculation of bacteria during *Nosema* challenges. We used a novel method, the suppression of *N. ceranae* with specific siRNAs, to measure the impacts of *Nosema* on the gut microbiome.

**Results:**

Suppressing *N. ceranae* led to significant positive effects on microbial abundance. Nevertheless, 15 bacterial taxa, including three core taxa, were negatively correlated with *N. ceranae* levels. In particular, one co-regulated group of 7 bacteria was significantly negatively correlated with *N. ceranae* levels.

**Conclusions:**

*N. ceranae* are negatively correlated with the abundance of 15 identified bacteria. Our results provide insights into interactions between gut microbes and *N. ceranae* during infection.

## Background

Animals evolve with their associated microorganisms as a unit and symbiotic microbes can facilitate the survival of hosts toward diverse stresses. When the host encounters pathogens, the microbial community responds and the dynamics can change dramatically [1]. As microbes can have varied effects on host challenges, the context of microbial function and host interactions is essential [2, 3]. In honey bees, eight core bacterial taxa have been identified. Collectively, these microbes have been shown to impact honey bee metabolism and immune responses towards infections, altering disease susceptibility [4–8].

*Nosema ceranae* is a unicellular fungal parasite which infects honey bee mid-gut epithelial cells. Infection starts from ingestion of *N. ceranae* spores. The proliferation cycle of *N. ceranae* is approximately 4 days, at which point a large number of offspring spores are released from infected cells [9]. *N. ceranae* infection negatively affects honey bee physiology, behavior and immune responses [10]. *N. ceranae* has spread globally and this agent is one of the factors implicated in honey bee colony collapses [11, 12]. A few gene silencing efforts have shown promise in reducing *N. ceranae* proliferation [13–15].

During *N. ceranae* proliferation, this parasite inevitably encounters the gut microbial community. Within the honey bee gut, bacteria are dominated by eight species/clusters. Three bacteria are within the Gram-negative phylum *Proteobacteria*, including *Gilliamella apicola*, *Frischella perrara* and *Snodgrassella alvi*. Two bacteria are within the Gram-positive phylum *Firmicutes*, including *Lactobacillus mellis*, *Lactobacillus kunkeei* and Lactobacillus Firm5 (including *L. helsingborgensis*, *L. melliventris* and *L. kimbladii*). One *Bifidobacterium* cluster within the phylum *Actinomycetes*, including *B. asteroids*, *B. actinocoloniiforme* and *B. bohemicum*. Two other species clusters are within phylum *Alphaproteobacteria* of *Bartonellaceae* and *Acetobacteraceae* [16]*.* Some of these taxa are likely to be involved in honey and pollen digestion, along with many low-frequency opportunistic microbes [2, 7, 17]. Gut microbes show diverse responses towards *N. ceranae* infection [18]. Consequently, it might be possible to control *N. ceranae* infection by regulating gut microbes, providing a long-lasting strategy to improve overall bee health. Quantifying the responses of microbes to *N. ceranae* infection is a critical step in understanding these interactions. This is challenging, because inoculating honey bees with *N. ceranae* spores generally leads to co-inoculation with associated gut bacteria. Even after several rounds of Percoll purification, a fraction of high-throughput sequence reads from purified spores can still be aligned to bacteria [19, 20]. So, predicting responses of honey bee gut microbes towards *N. ceranae* proliferation using bees challenged with *N. ceranae* and control bees can lead to artefacts.

The gene *Dicer* is a key component of the RNA induced silencing complex, which is involved in developmental and physiological gene regulation [21, 22]. We previously found that targeting the gene *Dicer* using siRNA interrupted *N. ceranae* proliferation [23, 24]. We used this insight to more accurately determine associations between *N. ceranae* infection and gut bacteria loads.

## Results

**Fig. 1:**
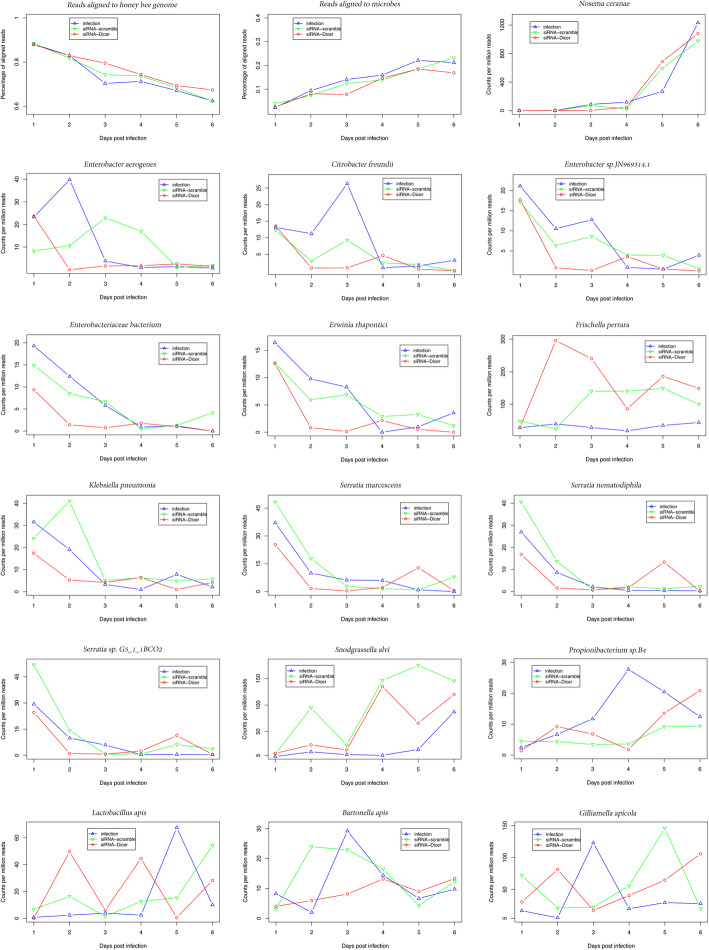
Read counts of microbes during the experimental period. Reads aligned to the bee genome decreased over time and the reads aligned to microbes increased. Overall, the microbes were negatively correlated with *N. ceranae* proliferation. X axis represents days post infection. Y axis represents the relative abundance of the studied microbes

As expected, sequence reads which aligned to the bee genome decreased while reads aligned to microbes increased gradually over the experimental period (Fig. 1) (File S1). The gene expression level of the *N. ceranae* RNA polymerase II small subunit (RPB1, KJ373285.1) was lower in the siRNA-Dicer group compared with the infection and siRNA-scramble groups at 3 dpi (Fig. 1). In total, 15 bacterial species/strains were found during the experimental period, including three core bacteria, *Snodgrassella alvi, Gilliamella apicola* and *Frischella perrara*. Two co-regulating bacterial groups were negatively correlated with *N. ceranae* over the entire experimental period. The marker genes of *Erwinia rhapontici*, *Enterobacteriaceae bacterium*, *Enterobacter sp. JN969314.1*, *Citrobacter freundii*, *Enterobacter aerogenes*, *Klebsiella pneumonia* and *Frischella perrara* were co-expressed (co-regulation group 1 of 7 bacteria) and significantly negatively correlated with *N. ceranae* RPB1 (corresponding correlation − 0.36, *P* = 0.03). Additionally, marker genes for *Serratia sp. G5_1_1BCO2*, *Serratia marcescens*, *Serratia nematodiphila* and *Snodgrassella alvi* were co-expressed (co-regulation group 2 of 4 bacteria), which was also negatively correlated with *N. ceranae* levels, even though this correlation was not statistically significant (corresponding correlation − 0.29, *P* = 0.08). *Bartonella apis*, *Propionibacterium sp. B4*, *Lactobacillus apis* and *Gilliamella apicola* did not cluster into any co-regulation group. None of these studied 15 microbes were statistically significantly differentially expressed at each day post-infection (T-test, *P* > 0.05). However, the co-regulated groups (*P* < 0.0001) and siRNA treatment (*P* < 0.0001) showed significant effects on specific microbe counts during the entire experimental period within the generalized linear model. Additionally, 6 bacteria in co-regulation group 1 (*E. rhapontici*, *E. bacterium*, *E. sp. JN969314.1*, *C. freundii*, *E. aerogenes*, *K. pneumonia*), 3 bacteria in co-regulation group 2 (*S. sp. G5_1_1BCO2*, *S. marcescens*, *S. nematodiphila*) and *G. apicola* were significantly enhanced towards siRNA treatment during the entire experimental period (*P* < 0.001).

## Discussion

When a parasite infects a host, infection success depends on host immune defenses, microbial community responses, and even competition from other parasites [25]. In turn, parasites spatially alter the relative abundance of gut microbes and other symbionts [26]. Honey bee gut microbes are important for food digestion and bee immune responses [17]. In honey bees, at least 406 bacterial species and variants have been found (based on the Holobee Bar database), including eight bacterial clusters that form the core gut microbial community [16, 17]. Within this core set of bacteria, the symbionts *Gilliamella apicola* and *Snodgrassella alvi* support food digestion and pathogen defenses, even though sequence variance may lead to functional variation [27, 28]. In contrast, the bacterium *Frischella perrara* triggers the honey bee melanization response [29]. Honey bees and their gut bacteria both respond to stressors and infection.

As two co-regulation groups were identified, the relative abundance of bacteria may not be independent of each other in the mid-gut tissue. Additionally, all of the studied bacteria were negatively correlated with *N. ceranae* proliferation, suggesting *N. ceranae* reduces bacterial abundance, at least for the studied 15 bacteria. In the gut microbiome, diet shapes bacterial diversity and responses towards *N. ceranae* infection, where a negative correlation between *S. alvi* and *N. ceranae* was found, which is consistent with our data [30]. When this microbial community was disturbed by antibiotics, honey bees were more susceptible towards *N. ceranae* and other opportunistic pathogens [8, 31]. Administration of *Lactobacillus* strains as food additives showed suppressive effects on *N. ceranae* spores [32].

Competition for limited food resources may lead to the decrease/exclusion of other competitors, as suggested by the competitive exclusion principle [33]. Therefore, a nutritional context is essential when interpreting any responses of the bacterial community toward parasite challenges. In our data, parasites negatively affected the relative abundance of all 15 studied gut bacteria, leading to interrupted food digestion and nutrient absorption. It remains unclear whether gut bacteria can actively defend the integrity of the gut tissue in bees. However, microbes can be regulated by diet to protect the gut integrity and support pathogen resistance in other organisms [34]. In prior work, honey bee pathogens have shown negative correlations with *S. alvi* [35, 36]. From our data, the levels of *F. perrara* and *S. alvi* were significantly enhanced with the reduction of *N. ceranae*. It remains unclear why *N. ceranae* negatively affected gut bacteria. Even though sugar was the only food resource in our studied bees, the chance is low that *N. ceranae* directly competes for this food resource with bacteria, as *N. ceranae* proliferates within epithelia cells and bacteria live in the lumen. Tissue destruction caused by infection might lead to the deterioration of the gut lumen, thus impacting bacteria.

Although microbial treatments provide a promising chemical-free strategy to control pathogen infection [37], caution is required, as improper probiotic supplements can lead to dysbiosis of gut microbes and susceptibility toward pathogens [38]. In our study, the relationship between *N. ceranae* and the studied microbes was correlative. However, the siRNA Dicer treatment and the reduced *N. ceranae* level was causative. We provided novel insights into interactions between gut parasites and other microbes over the entire life cycle of the parasite. Our study is limited to mid-gut tissues of newly-emerged workers. It will be interesting to screen *N. ceranae* and bacterial interactions in older forager bees, and in bees collected during different seasons.

## Conclusions

Levels of *N. ceranae* are negatively correlated with levels of gut bacteria. Gut bacteria were not independent with each other and were co-regulated. The relative abundance between the gut bacteria and *N. ceranae* was negatively correlated.

## Methods

### Ethics statement

Three apiaries (with 10, 10, and 20 colonies in each apiary, respectively) for bee sample collection were established at the USDA-ARS Bee Research Laboratory, Beltsville, Maryland, USA. No specific permits were required for the described studies. Studies involved the European honey bee (*Apis mellifera mellifera*), which is neither an endangered nor protected species.

### Infection and sample collection

*N. ceranae* spores were isolated from the mid-guts of one hundred heavily-infected honey bee workers from two colonies in the apiary. The abdomens of honey bee workers were homogenized using pestles in 500 mL distilled water, filtered through filtering mesh cloth (65 um pore size) and centrifuged 5 min at 3000 rpm. Spores were further purified using a Percoll gradient procedure [19]. Sealed brood frames were collected from multiple colonies and the brood frames were kept in an incubator to collect freshly emerged honey bee workers. Eighty newly-emerged workers were fed individually with 2 μL of sucrose solution as follows: (1) with 10^5^ *N. ceranae* spores without siRNA treatment, as the infection group; (2) with 10^5^ *N. ceranae* spores and 1.5 μg siRNA (targeting *N. ceranae* gene *Dicer)* as the siRNA-Dicer group; (3) with 10^5^ *N. ceranae* spores and 1.5 μg of a non-matching (scrambled) siRNA, as the siRNA-scramble group. The sequences of the designed siRNA were provided in a previous study [23]. Forty honey bees were kept in each rearing cup. In total, six rearing cups were constructed for three treatments, with two replicates per treatment. Five bees were sampled daily from 1 to 6 days post-infection (dpi) from each cup and ultimately pooled for RNA sequencing. After being anesthetized with CO_2_, the mid-gut tissue was dissected and the RNA was immediately extracted with TRizol [39]. Sequencing libraries were prepared and sequenced using the Illumina Hiseq 2000 platform. In total, 36 PCR-free Illumina RNA paired-end (151 nucleotide per read) libraries of mid-gut samples were sequenced (two replicates per treatment per day), at the University of Maryland Institute for Genomic Studies.

### Bioinformatics and microbial quantification

On average, 58 million reads (114 nucleotides for each paired-read member) were generated from each library, after trimming and quality control using FastQC (https://www.bioinformatics.babraham.ac.uk/projects/fastqc/) and SEQTK (https://github.com/lh3/seqtk). Sequencing reads were aligned to the honey bee genome assembly (Amel_HAv3.1) with the hisat2 package under default settings [40]. Reads that matched the honey bee genome were removed. On average, 14,258,564 remaining reads per library were then aligned to the Holobee ‘Bar’ database and 7,260,989 reads per library were mapped (>138X coverage of the database) including *N. ceranae* and other bee-associated microbes. The Holobee Bar database is a curated resource for microbes associated with honey bees (https://data.nal.usda.gov/dataset/holobee-database-v20161). In order to remove false-positive microbial classifications and restrict reads to the more prevalent bacteria responding towards the parasite infection, microbes found in fewer than 22 libraries of the sequencing libraries were removed. The counts were normalized using the weighted trimmed mean of M-values (TMM). Two replicates of each treatment for each post-infection day were used to calculate the variance in order to identity significantly regulated bacteria with edgeR [41]. The *P* values were corrected for multiple comparisons using false discovery rate (FDR). Significantly differentially expressed taxa met three criteria: (1) the relative abundance of the bacterium is significantly different between the infection group and the siRNA-Dicer group; (2) the relative abundance of the bacterium is significantly different between the siRNA-Dicer group and the siRNA-scramble group; and (3) the relative abundance of the bacterium is not significantly different between the siRNA-scramble group and the infection group.

### Statistical analysis

Significantly differentially expressed taxa were tested using t-tests at each time point and the *P* value was adjusted for multiple comparisons with FDR using R (version 3.5.1). In order to analyze bacterial patterns over the infection period, co-regulation between *N. ceranae* and bacteria clusters was analyzed using the WGCNA package [42]. The normalized read counts of bacteria were treated as the genotype and counts of *N. ceranae* were treated as phenotype. To further test the effect of siRNA treatment on the microbe enrichment, a generalized linear model was performed using R (version 3.5.1). Within the linear model (counts ~ treatment + day + cage + group + microbe), counts indicate normalized microbe counts. Treatment indicates siRNA-Dicer group, siRNA-scramble group and infection group. Day indicates number of days post infection. Cage indicates each of the two cage replicates. Group indicates co-expression groups obtained from WGCNA analysis. Microbe indicates each of 15 microbe species. The code is provided in supplementary file S2.

## Supplementary information

**Additional file 1 File S1** sequencing alignment statistics and normalized microbe counts.

**Additional file 2 File S2** code used for the statistics.

## Data Availability

The raw sequencing reads are deposited in NCBI bio-project PRJNA399493.

## Abbreviations

siRNA: small interfering RNA
PRB1: RNA polymerase II small subunit
WGCNA: Weight Correlation Network Analysis
