# Supplementary material for: Targeting the honey bee gut parasite Nosema ceranae with siRNA positively affects gut bacteria
Source: BMC Microbiol. 2020 Aug 17;20:258. doi: 10.1186/s12866-020-01939-9 (PMC7433167; doi:10.1186/s12866-020-01939-9)
Supplement: Supplementary file 2 — Additional file 2 File S2 code used for the statistics. [file 12866_2020_1939_MOESM2_ESM.docx]

***Nosema ceranae* proliferation negatively affected honey bee gut bacteria**

Qiang Huang^1*^, Jay D. Evans^2*^

^1^ Honeybee Research Institute, Jiangxi Agricultural University, Zhimin Avenue 1101, 330045, Nanchang, China

^2^ USDA-ARS Bee Research Laboratory, BARC-East Building 306, Beltsville, Maryland, 20705, USA

^*^ For correspondence

Email: qiang-huang@live.com (QH), [Jay.Evans@ARS.USDA.GOV](javascript:main.compose('new',%20't=%22Evans%2C%20Jay%22%20%3CJay.Evans%40ARS.USDA.GOV%3E')) (JDE);

**Material and method**

1. Code used for edgeR

library("limma")

library("edgeR")

data_parasite <- read.table("raw_counts.txt",header=TRUE)

counts_parasite <- data_parasite[,2:37]

rownames (counts_parasite) <- data_parasite[,1]

dim (counts_parasite)

group_parasite <- c(rep("D1_inf",2),rep("D1_scrum",2),rep("D1_si",2),rep("D2_inf",2),rep("D2_scrum",2),rep("D2_si",2),rep("D3_inf",2),rep("D3_scrum",2),rep("D3_si",2),rep("D4_inf",2),rep("D4_scrum",2),rep("D4_si",2),rep("D5_inf",2),rep("D5_scrum",2),rep("D5_si",2),rep("D6_inf",2),rep("D6_scrum",2),rep("D6_si",2))

counts_parasite <- DGEList(counts=counts_parasite,group=group_parasite)

Nor <- calcNormFactors(counts_parasite,method="TMM")

Nor.count <- cpm(Nor)

Nor_dispersion <- estimateCommonDisp(Nor)

design <- model.matrix(~0 + group_parasite)

y <- estimateDisp(Nor,design,robust=TRUE)

y$common.dispersion

Nor_dispersion$common.dispersion

Raw_pvalue <- exactTest(Nor,dispersion=0.01,pair=c("D1_inf","D1_si"))

Adjusted_pvalue <- p.adjust(Raw_pvalue$table$PValue,method="BH")

2. code for generalized linear model (in R)

mode5 <- lm(counts~treatment+Day+cage+group+microbe,data=data)

3. code for WGCNA (In R)

library("WGCNA")

options(stringsAsFactors = FALSE)

mRNAdata = read.csv("genotype.csv")

datExpr_Ncer = as.data.frame(t(mRNAdata[, 2:37]))

names(datExpr_Ncer) = mRNAdata$genotype

rownames(datExpr_Ncer) = names(mRNAdata)[2:37]

gsg = goodSamplesGenes(datExpr_Ncer, verbose = 3)

gsg$allOK

if (!gsg$allOK)

{

# Optionally, print the gene and sample names that were removed:

if (sum(!gsg$goodGenes)>0)

printFlush(paste("Removing genes:", paste(names(datExpr0)[!gsg$goodGenes], collapse = ", ")));

if (sum(!gsg$goodSamples)>0)

printFlush(paste("Removing samples:", paste(rownames(datExpr0)[!gsg$goodSamples], collapse = ", ")));

# Remove the offending genes and samples from the data:

datExpr0 = datExpr0[gsg$goodSamples, gsg$goodGenes]

}

sampleTree = hclust(dist(datExpr_Ncer), method = "average")

sizeGrWindow(12,9)

par(cex = 0.6)

par(mar = c(2,6,2,2))

plot(sampleTree, main = "",sub="", xlab="", cex.lab = 1.6,

cex.axis = 1.6, cex=1.5, cex.main = 2)

traitData = read.csv("phenotype.csv")

dim(traitData)

names(traitData)

allTraits = traitData

names(datExpr_Ncer)

NcerSamples = rownames(datExpr_Ncer)

traitRows = match(NcerSamples, allTraits$Phenotype);

datTraits = allTraits[traitRows, -1];

rownames(datTraits) = allTraits[traitRows, 1];

collectGarbage();

# Re-cluster samples

sampleTree2 = hclust(dist(datExpr_Ncer), method = "average")

# Convert traits to a color representation: white means low, red means high, grey means missing entry

traitColors = numbers2colors(datTraits, signed = FALSE);

# Plot the sample dendrogram and the colors underneath.

sizeGrWindow(12,9)

par(cex = 0.6)

par(mar = c(20,500,20,20)+0.1)

plotDendroAndColors(sampleTree2, traitColors,groupLabels = names(datTraits),

main = "Sample dendrogram and trait heatmap")

save(datExpr_Ncer, datTraits, file = "mRNA_Ncer.RData")

lnames = load(file = "mRNA_Ncer.RData")

lnames

powers = c(c(1:10), seq(from = 12, to = 20, by=2))

sft = pickSoftThreshold(datExpr_Ncer, powerVector = powers, verbose = 5,blockSize = 1)

sizeGrWindow(9, 5)

par(mfrow = c(1,2))

cex1 = 0.9

plot(sft$fitIndices[,1], -sign(sft$fitIndices[,3])*sft$fitIndices[,2],

xlab="Soft Threshold (power)",ylab="Scale Free Topology Model Fit,signed R^2",type="n",

main = paste("Scale independence"));

text(sft$fitIndices[,1], -sign(sft$fitIndices[,3])*sft$fitIndices[,2],

labels=powers,cex=cex1,col="red")

abline(h=0.80,col="red")

plot(sft$fitIndices[,1], sft$fitIndices[,5],

xlab="Soft Threshold (power)",ylab="Mean Connectivity", type="n",

main = paste("Mean connectivity"))

text(sft$fitIndices[,1], sft$fitIndices[,5], labels=powers, cex=cex1,col="red")

net = blockwiseModules(datExpr_Ncer, power = 5,

TOMType = "unsigned", minModuleSize = 2,

reassignThreshold = 0, mergeCutHeight = 0.25,

numericLabels = TRUE, pamRespectsDendro = FALSE,

saveTOMs = TRUE,

saveTOMFileBase = "Ncer_TOM",

verbose = 3)

table(net$colors)

sizeGrWindow(12, 9)

mergedColors = labels2colors(net$colors)

plotDendroAndColors(net$dendrograms[[1]], mergedColors[net$blockGenes[[1]]],

"Module colors",

dendroLabels = FALSE, hang = 0.03,

addGuide = TRUE, guideHang = 0.05)

moduleLabels = net$colors

moduleColors = labels2colors(net$colors)

MEs = net$MEs;

geneTree = net$dendrograms[[1]];

save(MEs, moduleLabels, moduleColors, geneTree,

file = "networkConstruction_auto_Ncer.RData")

lnames=load(file="networkConstruction_auto_Ncer.RData")

nGenes = ncol(datExpr_Ncer);

nSamples = nrow(datExpr_Ncer);

MEs0 = moduleEigengenes(datExpr_Ncer, moduleColors)$eigengenes

MEs = orderMEs(MEs0)

moduleTraitCor = cor(MEs, datTraits, use = "p");

moduleTraitPvalue = corPvalueStudent(moduleTraitCor, nSamples)

write.csv(moduleTraitPvalue,file="train.csv")

sizeGrWindow(15,13)

textMatrix = paste(signif(moduleTraitCor, 2), "\n(",

signif(moduleTraitPvalue, 1), ")", sep = "");

dim(textMatrix) = dim(moduleTraitCor)

par(mar = c(6, 10.5, 3, 3))

labeledHeatmap(Matrix = moduleTraitCor,

xLabels = names(datTraits),

yLabels = names(MEs),

ySymbols = names(MEs),

colorLabels = FALSE,

colors = blueWhiteRed(50),

textMatrix = textMatrix,

setStdMargins = FALSE,

cex.text = 0.4,

zlim = c(-1,1),

main = paste("Module-trait relationships"))

names(datExpr_Ncer)

annot = read.csv("annotation.csv")

dim(annot)

names(annot)

probes=names(datExpr_Ncer)

geneInfo = data.frame(moduleColor = moduleColors,

Probe = probes,

moduleLabels =moduleLabels

);

write.csv(geneInfo, file = "data_Ncer.csv")

**Results:**

1. generalized linear model

summary(mode5)

Call:

lm(formula = counts ~ treatment + Day + cage + group + microbe,

data = data)

Residuals:

Min 1Q Median 3Q Max

-105.45 -12.00 -5.21 5.83 375.92

Coefficients: (1 not defined because of singularities)

Estimate Std. Error t value Pr(>|t|)

(Intercept) 151.4427 25.9573 5.834 9.50e-09 ***

treatmentinfection -10.4861 4.3855 -2.391 0.0172 *

treatmentscramble 0.7672 4.3855 0.175 0.8612

Day 0.6393 1.0483 0.610 0.5423

cage 5.8285 3.5807 1.628 0.1042

group -49.2278 9.8063 -5.020 7.10e-07 ***

microbeCitrobacter_freundii -104.0972 16.9850 -6.129 1.75e-09 ***

microbeEnterobacter_aerogenes -100.8944 16.9850 -5.940 5.21e-09 ***

microbeEnterobacter_sp -103.6833 16.9850 -6.104 2.02e-09 ***

microbeEnterobacteriaceae_bacterium -104.9611 16.9850 -6.180 1.30e-09 ***

microbeErwinia_cf_rhapontici -105.0528 16.9850 -6.185 1.26e-09 ***

microbeFrischella_perrara -11.1111 16.9850 -0.654 0.5133

microbeGilliamella_apicola 38.6333 9.8063 3.940 9.27e-05 ***

microbeKlebsiella_pneumoniae -99.3639 16.9850 -5.850 8.69e-09 ***

microbeLactobacillus_cf_apis 6.4083 9.8063 0.653 0.5137

microbePropionibacterium_s -2.0833 9.8063 -0.212 0.8318

microbeSerratia_marcescens -50.5972 9.8063 -5.160 3.52e-07 ***

microbeSerratia_nematodiphila -53.2556 9.8063 -5.431 8.62e-08 ***

microbeSerratia_sp -51.5611 9.8063 -5.258 2.13e-07 ***

microbeSnodgrassella_alvi NA NA NA NA

---

Signif. codes: 0 ‘***’ 0.001 ‘**’ 0.01 ‘*’ 0.05 ‘.’ 0.1 ‘ ’ 1

Residual standard error: 41.6 on 521 degrees of freedom

Multiple R-squared: 0.3035, Adjusted R-squared: 0.2794

F-statistic: 12.61 on 18 and 521 DF, p-value: < 2.2e-16
